# Inclusion of phenolic bioactives in high amylose corn starch for gastro-intestinal delivery

**DOI:** 10.3389/fnut.2022.981408

**Published:** 2022-08-25

**Authors:** Hila Tarazi Riess, Carmit Shani Levi, Uri Lesmes

**Affiliations:** Laboratory of Chemistry of Foods and Bioactives, Department of Biotechnology and Food Engineering, Technion – Israel Institute of Technology, Haifa, Israel

**Keywords:** capsaicin, curcumin, delivery, digestibility, inclusion complexes, starch

## Abstract

Starch is a staple food component with intricate architectures, some of which can be utilized as polysaccharidic delivery vehicles for bioactive compounds. This work describes the use of high amylose corn starch (HACS) to fabricate V-amylose inclusion complexes entrapping capsaicin or curcumin. In line with past studies, X-ray diffraction, differential scanning calorimetry, static laser scattering and scanning electron microscopy help affirm the formation of V6III-type complexes. Such HACS complexes entrap capsaicin and curcumin in structures with higher levels of crystallinity compared to HACS alone (14.61 ± 0.08%, 14.65 ± 0.08% vs. 10.24 ± 0.24%, respectively), high levels of encapsulation efficiency (88.77 ± 5.7% and 66.3 ± 0.99%, respectively) but without significant differences in colloid sizes between the various inclusion complexes (58.25 ± 1.34 μm or 58.98 ± 2.32 μm, respectively). In turn, *in vitro* gastro-intestinal digestion of HACS complexes with capsaicin or curcumin revealed both, phenolic bioactives significantly (*p* < 0.05) attenuated the intestinal breakdown of HACS. Interestingly, this attenuated HACS digestibility was accompanied by high gastric retention of the payloads and their sustained release during 2 h of exposure to intestinal conditions. Altogether, this work presents starch-based delivery systems that can entrap phenolic bioactives, release the payload in the intestine and possibly attenuate starch breakdown (because of its increased crystallinity). Thus, this work offers a platform for infusing foods with bioactive phenolics and stall the breakdown of starch.

## Introduction

The rise in life expectancy and the prevalence of non-communicable diseases (e.g., obesity and diabetes) stimulate efforts to produce functional food solutions with extra-nutritional values (1). Such added-values can stem from the adequate provision of non-nutrient bioactives with demonstrated health benefits, such as resveratrol, lutein, EGCG, curcumin and capsaicin (2). However, such compounds may not be sufficiently and/or widely present in common diets, which mandate exploring avenues for their effective delivery to the consumer. To this end, enrichment of such bioactives into accessible staple foods, e.g., bread and pasta, is a scientific and technological challenge. Such delivery hurdles range from problematic sensorial perception (e.g., bitter taste of EGCG) to poor techno-functionality (e.g., poor chemical stability and/or poor solubility of curcumin) and low bioavailability. Therefore, one can note the adoption of a pharmaceutical approach in the development of food-grade delivery systems that encapsulate, protect and deliver bioactive components (3, 4). Such systems are required to comply with the food matrix properties and stability under processing and storage conditions while enabling controlled spatio-temporal release of the cargo along the gastrointestinal (GI) tract (3, 4).

Food delivery systems can be formed from food proteins, lipids, carbohydrates or mixtures thereof (3–5). In this respect, starch is a staple and affordable macronutrient which has diverse architectures, some have been shown apt to serve for encapsulation of food bioactives (6). In fact, starch is a family of polysaccharides that are non-toxic, widely available, inexpensive, easy to use and diversely digestible (7). Current literature discloses evidence of three classes of starch according to their digestibility: rapidly digestible starch (RDS), slowly digestible starch (SDS), and resistant starch (RS) (8–10). Starch digestibility is influenced by structural characteristics such as granule size, amylopectin:amylose ratio, mean molecular weight and degree of crystallinity. However, this is an open field of ongoing research which mainly reports the qualitative links between the parameters and the observed effects (9, 10). Chemically, starch is identified as a mixture of two classes of glucose-based polysaccharides: amylopectin and amylose (9). Amylopectin is a branched glucose-based polysaccharide in which glucose repeating units are linked by α-(1→4) and α-(1→6) linkages contrary to amylose which is predominantly a linear form of α-(1→4) linked glucose units (9). Out of which, amylose has been documented to form supra-molecular inclusion complexes, termed as V-type amylose (11).

The V-form of amylose is a single-chain left-handed helix with a large cavity, where the ligand can be situated, either inside the helix cavity, between the helices or in both sites. Molecular studies show the ligand delineates the number of glucosyl residues per helical turn (6, 7, or 8) (12–15). The helix is stabilized by intramolecular interactions (such as Van der Waals forces and hydrogen bonds) between the turns along the helix, and by intermolecular forces that govern the interaction between amylose and its entrapped moiety (11). The interaction of amylose with the ligand decreases its solubility, increases gelatinization temperature, delays retrogradation, and increases its resistance to digestive enzymes (16). Thus, there are evidence that amylose inclusion complexes can be utilized as delivery vehicles that encapsulate various bioactive compounds and direct their release and bioavailability in the GI (17).

High corn starch amylose (HACS) has been shown to be a good commercial alternative to fabricate V-type inclusion complexes (17–19). In fact, studies show that HACS may form functional nano-capsules that can be useful controlled delivery systems for lipophilics, such as genistein and capsaicin (19–24). Yet, further evidence is needed to improve our understanding of their digestive fate and the possible implications to consumer health. This study sought to extend such studies and test the hypothesis that different phenolic bioactives can be encapsulated using HACS to yield powders that are digestible and sustain the release of the entrapped payload in the gastro-intestine. Specifically, the current study opted to focus on the bioactives capsaicin (CAP) and curcumin (CUR) which are natural phytochemicals with attributed health benefits (21, 25–31) alongside challenging sensorial and physicochemical attributes (e.g., pungency and poor solubility) (32, 33). Thus, this work extends recent work (24) and offers new evidence on the formation and digestive fate of amylose inclusion complexes with the lipophilic compounds.

## Materials and methods

### Materials

High-amylose corn starch (HACS) was purchased from National Starch (HYLON VII; Bridgewater, NJ, United States). Capsaicinoids (Batch# FZSWCAP171214, ≥95%) and curcuminoids (Batch# FZ180817, ≥95%) were purchased from FZBIOTECH (Xi’an Fengzu Biological Technology Co., Ltd., China). The following materials were purchased from Sigma-Aldrich (Rehovot, Israel): salivary α-amylase from *Aspergillus oryzae* (A1031, Lot# BCCB7689, 1.52 ± 0.08 U/mg); pepsin from porcine gastric mucosa (P7000, Lot#BCCF8993, 383 ± 22 U/mg); pancreatic α-amylase from porcine pancreas (A3176, Lot# SLCG7434, 10.14 ± 0.72 U/mg); trypsin from porcine pancreas (T0303, Lot# SLCB7350, 69.5 ± 5.5 U/mg); α-chymotrypsin from bovine pancreas (C4129, Lot# SLBT5554, 27.8 ± 2.9 U/mg); all were tested for their activity in-house, as described (34). Phenylmethanesulfonyl fluoride (PMSF) solution, taurocholic acid sodium salt hydrate (Lot# SLBL1190V, ≥95%); HPLC analytical grade ethanol (99.8%); HPLC analytical grade methanol (99.9%); HPLC analytical grade water; HPLC analytical grade acetonitrile (99.8%) were also purchased from Sigma-Aldrich (Rehovot, Israel). Glycodeoxycholate acid sodium salt (≥97%) was obtained from Holland Moran (Yahud, Israel). Ethyl-acetate was purchased from Gadot-Group (Netanya, Israel). Simulated saliva, gastric and duodenal fluids were prepared according to INFOGEST protocol (34, 35). All reagents used were of analytical grade and all solutions were prepared with double distilled water (DDW).

### Methods

#### Preparation of high amylose corn starch-phenol inclusion complexes

Complexation was prepared by the acidification of an alkali solution as previously described (24) with slight modifications for the entrapment of curcumin. One gr of HACS was dissolved in 200 ml 0.1 N KOH solution (pH 13) at 90°C for 1 h, then cooled to 30°C. Concomitantly, 100 mg of CAP or CUR were dissolved separately in 20 ml 0.1 N KOH or pure absolute ethanol at 30°C for 15 min, respectively. Starch and cargo solutions were mixed together (for HACS-CUR complex, HACS solution adjust to pH 7 before mixing) and the mixture was induced toward complexation and precipitation by adjusting the pH to 4.7 (± 0.2) using 4% H_3_PO_4_ while kept at 30°C and gentle mixing (100 rpm in an incubator shaker, TU-400 Orbital Shaker Incubator, MRC, Holon). The precipitate was collected by centrifugation (6,000 *g*, 20 min, 10°C) and washed three times with 50% ethanol/water mixture (v/v) to remove loosely bound cargo. The complexes were then freeze-dried, pulverized into a fine powder and kept in desiccators until further analysis.

#### Characterization of molecular complexes

##### X-ray diffraction

The crystallographic x-ray diffraction patterns of the complexes were determined using a SmartLab 3 kW X-ray diffractometer (Rigaku, Japan). The X-ray source was a copper X-ray tube (CuKα1; λ = 1.54 Å) operating at 40 kV and 30 mA. The X-ray source employed was a copper X-ray tube (CuKα1; lambda = 1.54 Å) operating at 40 kV and 30 mA. Diffraction patterns were acquired at 2θ diffraction angles of 5–30° at 1.2°min-1 scanning rate with a 0.5° scattering slit, a 0.17° divergence slit, and a 12 mm receiving slit. The crystallinity degree [%] of the samples was calculated using OriginPro 2022b according to the following equation (36):


$$\%\mathrm{crystallinity}=\frac{c\,r\,y\,s\,t\,a\,l\,l\,i\,n\,e\,a\,r\,e\,a}{o\,v\,e\,r\,a\,l\,l\,a\,r\,e\,a}\times 100$$

##### Differential scanning calorimetry

Differential scanning calorimetry thermograms of HACS-phenol complexes were obtained using a DSC calorimeter (DSC250, TA Instruments, United States). Pre-weighed dried powder (approximately 2–3 mg) was weighed into aluminum pan and carefully sealed. An empty aluminum pan was used as a reference. The samples were analyzed between 10 and 250°C, at a heating rate of 5°C/min and N_2_ flow rate of 50 ml/min. Data analyses for all measurements were performed using TRIOS software (TA Instruments, DE, United States).

##### High-resolution scanning electron microscopy

The microstructure and surface morphology of the lyophilized complexes were examined by a high-resolution scanning electron microscopy (Zeiss Ultra-Plus FEG-SEM) located in the Technion’s Electron Microscopy Center (MIKA). The micrographs were obtained at voltage of 3 kV, aperture size of 30 μm, in-lens and SE2 detectors. Prior to measurements the dried powders were glued on a carbon tape and then coated with carbon.

##### Particle size distribution and volume mean diameter (d_4,3_)

The particle size of suspensions produced by the complexation procedure was determined using a laser diffraction particle size analyzer (Malvern Mastersizer 3000, Malvern Instruments Ltd., Malvern, Worcestershire, United Kingdom) equipped with a wet sample dispersion unit (Malvern Hydro MV, United Kingdom). The optical properties were defined as a refractive index of 1.437 (starch) and 1.33 (dispersant KOH) and an absorption index of 0.001. In the sample port, the samples were dispersed in distilled water at 1,200 rpm until an obscuration of 4–8% was obtained. All measurements were performed in triplicate with every sample measured thrice to ensure consistency and reproducibility of results.

#### Ligand content in the complexes

The ligand content of the complexes was determined on the basis of the ligand released following full amylolytic hydrolysis with pancreatic amylase. Dried complex sample (15 mg) was incubated in 1 ml of pancreatic amylases (140 units/ml enzymatic activity, pH 6.9, 37°C) for 24 h with slow rotation (20 rpm) on a horizontal tube rotator. Following incubation, the samples were centrifuged at 16,000 × *g* for 15 min. The supernatant was mixed with ethyl acetate (1:1 v/v) and vigorously mixed for 30 min. Then, the ethyl acetate phase was separated, kept in a hood overnight for complete solvent evaporation, re-dissolved in absolute ethanol for quantification using RP-HPLC method (see section “Quantitation of curcumin or capsaicin by RP-HPLC”).

Overall, the experiments were performed three independent repetitions.

The loading capacity (LC) and encapsulation efficiency were calculated according to the following

equations (37):


$$LC\left[\%\right]=\frac{p\,h\,e\,n\,o\,l\,i\,n\,t\,h\,e\,c\,o\,m\,p\,l\,e\,x\,\left[m\,g\right]}{c\,o\,m\,p\,l\,e\,x\,w\,e\,i\,g\,h\,t\,\left[m\,g\right]}\times 100\%$$

$$EE\left[\%\right]=\frac{p\,h\,e\,n\,o\,l\,i\,n\,t\,h\,e\,c\,o\,m\,p\,l\,e\,x\,\left[m\,g\right]}{i\,n\,i\,t\,i\,a\,l\,a\,m\,o\,u\,n\,t\,o\,f\,p\,h\,e\,n\,o\,l\,\left[m\,g\right]}\times 100\%$$

#### Quantitation of curcumin or capsaicin by RP-HPLC

Quantitation of extracted phenols was made by a reverse-phase HPLC (Agilent Technologies, Wilmington, DE, United States) equipped with a UV detector. Briefly, 20 μl injection volume was separated by a C18 column (ZORBAX Eclipse Plus, 95 Å, 4.6 × 250 mm, 5 μm, 959990-902) using isocratic elution program. CUR was detected at 425 nm and eluted with a mobile phase containing acetonitrile–water–acetic acid (50:49:1 v/v/v) at a flow rate of 1 ml/min and 20°C. CAP was UV detected at a wavelength of 281 nm and eluted using with a mobile phase containing methanol and water (80:20 v/v) at a flow rate of 1 ml/min and 20°C. Calibration curves was compiled based on seven samples of standard solutions at varying concentrations (*R*^2^ = 0.9987), so as to enable quantification of CUR or CAP.

#### Evaluating the digestive performance of high amylose corn starch-phenol complexes

##### Semi-dynamic *in vitro* digestion (IVD)

Semi-dynamic *in vitro* digestion experiments were conducted in accordance to the international consensus INFOGEST protocol for research on food digestion and its relevant adjustments (34, 35, 38). The experiments recreating adult gastro-intestinal digestion were performed in a dual auto titration unit (Titrando 902, Metrohm, Switzerland) equipped with a water jacketed reactor maintained at 37°C, stirred at a constant rate of 250 rpm. TIAMO 2.0 software was used to generate pH gradients in the IVD model based on updated physiological data that is bio-relevant to food research (39–41).

Briefly, samples of 60 mg of HACS-phenol complex, empty complex (HACS control) or physical mixture were mixed for 30 s with simulated salivary fluid (SSF), salivary α-amylase (final concentration of 75 U/ml) and CaCl_2_ (final concentration of 1.5 mM in SSF) to form an oral bolus of 50 ml (1.2 mg/ml). Then, the bolus was mixed with 45 ml preheated simulated gastric fluid (SGF) (pH 1.3, 37°C), followed by the addition of 5 ml pepsin (final concentration of 2,000 U/ml) and CaCl_2_ (final concentration of 0.15 mM in SGF). The pH was rapidly adjusted to pH 4.5 (using 5 M NaOH) and a computer-controlled gastric pH gradient was initialized (by auto titration of 0.3 M HCl) for a 2 h gastric phase. Subsequently, 50 ml of gastric effluent was aspirated and mixed with an equal volume of simulated duodenal fluid (SDF), pancreatic α-amylase (200 U/ml), trypsin (100 U/ml), α-chymotrypsin (25 U/ml), bile salts (5 mM sodium glycodeoxycholate and 5 mM taurocholic acid sodium salt hydrate) and CaCl_2_ (final concentration of 0.6 mM in SDF). The intestinal phase was initiated and maintained at pH 6.25 (using 0.3 M NaOH) for 2 h.

During digestion, 10 aliquots of 1.5 ml were aspirated as follows: one after the oral phase, three samples from the gastric phase at 30, 60, and 120 min and six samples from the intestinal phase at 10, 20, 30, 60, 90, and 120 min. Aspirates were rapidly inactivated using 1 M NaOH to reach pH 7 for gastric samples, or PMSF (final concentration of 0.5 mM PMSF) for intestinal samples and placed on ice until centrifugation at 16,000 *g* for 15 min. The supernatant was stored at −20°C for further analyses.

##### Starch degradation evaluation by dinitrosalicylic (DNS) acid assay

Reducing sugars released during *in vitro* digestion by α-amylase were measured using the dinitrosalicylic acid (DNS) method as recommended by the INFOGEST protocol (34, 35). For this, 1% (w/v) maltose stock was used to form various concentrations of maltose to be used to determine calibration curve (*R*^2^ = 0.9995). The digesta samples were diluted (1:3 v/v) with distilled water and 120 μl of the resulting solution were mixed with 120 μl of 1% (w/v) DNS reagent (consisting of 1 g 3,5-dinitrosalicylic acid, 0.05 g sodium sulfite and 1 g sodium hydroxide in 100 ml distilled water). Maltose calibration samples were treated similarly with DNS reagent. The mixture was then vortexed and incubated at 95°C for 10 min to allow the red-brown color development. Next, each sample was mixed with 31 μl Rochelle salt (40% potassium sodium tartrate solution) to stabilize the color. After sample cooling to room temperature, 200 μl of sample were loaded into a 96-well plate and the absorbance at 575 nm was recorded. Values were subsequently transposed to maltose concentrations according to the calibration curve.

#### Data collection and statistical analyses

All tests were performed at least in triplicate and the data were expressed as mean ± standard deviation (SD). The significant differences between groups were tested by *t*-test analysis using GraphPad Prism (version 9.3.1). A significance level was considered to be *p* < 0.05.

## Results and discussion

Delivery of lipophilic bioactives using staple food polysaccharides, namely starch, can be achieved through deliberate formation of HACS inclusion complexes. This study uses CUR and CAP as model phenolic bioactives that are lipophilic and can be successfully entrapped and released by HACS inclusion complexes. The underling hypothesis of this study was that the different bioactives induce formation of various structures of V-amylose that in turn affect their susceptibility to hydrolysis by digestive conditions (i.e., acidity and enzymes).

### Characterization of molecular complexes

#### X-ray diffraction

**FIGURE 1:**
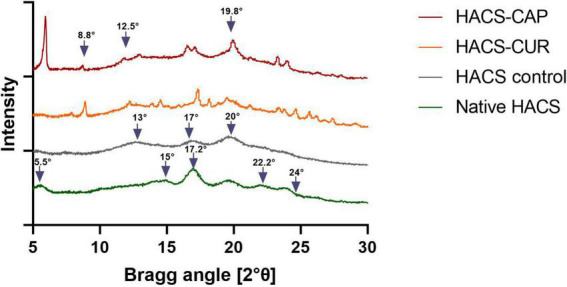
X-ray diffraction patterns of HACS-CAP, HACS-CUR, HACS control (empty) and native HACS.

Studies show that the formation of V-amylose is typically accompanied by distinct X-ray diffraction patterns and/or thermal behavior changes noticeable by DSC (11, 42, 43). Thus, X-ray diffraction patterns of HACS-phenol inclusion complexes, HACS control (HACS processed similarly in the absence of guest molecules) and native HACS (raw HACS as received from the manufacturer) were recorded and are shown in Figure 1. The characteristic V-type patterns with peaks at Bragg angles of 2θ = 13°, 17°, 20° were recorded for processed HACS, HACS-CUR and HACS-CAP, concurring with previous reports (19, 44). These findings substantiate a process-induced transition of HACS from B-type in native HACS (peaks at 2θ = 5.5°, 15°, 17.2°, 22.2°, and 24°) to the functional V-type (24, 45). The HACS-CUR and HACS-CAP complexes had additional peaks at 8.8°, 12.5°, and 19.8° further supporting the formation of a V6III type structure in which each helical turn comprises of six glucose residues and the overall starch particulate assembly comprises of crystalline regions interrupted by amorphous regions (12, 24, 46, 47). X-ray diffraction patterns of pure CAP and pure CUR are presented in Supplementary material. Calculations of the crystallinity degree of HACS-phenol inclusion complexes summarized in Table 1 indicate that the presence of guest molecules the well-ordered crystalline regions grew at the expense of the less ordered amorphous ones. This seems to support the overall paradigm that inclusion of lipophilic compounds induces the formation of V-type structures that tend to form well-ordered regions (12, 47).

**TABLE 1:** Crystallinity degree (*n* = 3) of HACS-phenol complexes, HACS control (empty) and native HACS.

Sample	Crystallinity degree [%, ± SD]
HACS-CUR	14.61 ± 0.08 ^a^
HACS-CAP	14.65 ± 0.08 ^a^
HACS control	10.24 ± 0.24 ^b^
Native HACS	9.76 ± 0.06 ^c^

^a–c^Statistically different values (p < 0.05).

#### Differential scanning calorimetry

**FIGURE 2:**
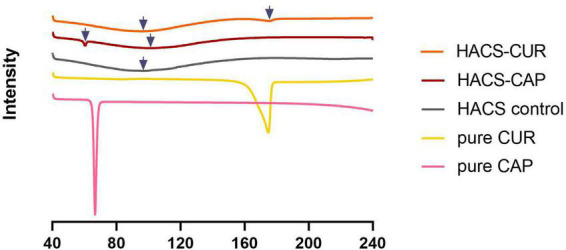
DSC thermograms of HACS-CUR, HACS-CAP, HACS control (empty), pure CUR and pure CAP.

Formation of V-type inclusion complexes also hinders the thermal behavior of the polysaccharides (11, 43). Heating of HACS-phenol inclusion complexes induces their dissociation which typically produces endotherms with peak temperatures in the range from 80 to 120°C, according to the complex structure (48, 49). In addition, the dissociation enthalpy is relative to the amount of the entrapped ligand in the complex (50). Thus, DSC thermograms of HACS-CUR, HACS-CAP and controls (pure CUR, pure CAP, HACS control) were determined and are shown in Figure 2. These findings disclose endothermic peaks and enthalpies obtained for HACS-CAP (100.95 ± 0.06°C, ΔH = 239.31 ± 1.89 J/g) and HACS-CUR (96.68 ± 1.86°C, ΔH = 227.12 ± 0.04 J/g). The thermogram of HACS control shows an endothermal peak at 96.7 ± 0.06°C, and an enthalpy of Δ*H* = 219.93 ± 0.4 J/g, which is significantly lower than the HACS-phenol complexes. Significant differences (*p* < 0.05) are noted between the enthalpies of HACS-CAP and HACS-CUR (ΔH = 239.31 ± 1.89 J/g and ΔH = 227.12 ± 0.04 J/g, respectively) which are indicative of differences in the ratio of ligand entrapped within the complex. In addition, weak endothermic peaks were observed at 60.31 ± 0.01°C and 175.46 ± 0.06°C in HACS-CAP and HACS-CUR inclusion complexes, respectively. DSC curves obtained for pure CAP or CUR give acuminate single peaks at 66.63 ± 0.01°C and 174.56 ± 0.02°C with an enthalpy of Δ*H* = 73.85 ± 1.15 J/g and Δ*H* = 124.97 ± 0.69 J/g, respectively. This concurs with previous values reported in the literature (51, 52) and suggest the additional peaks observed in HACS-CAP and HACS-CUR arise from some non-complexed CAP or CUR.

#### High-resolution scanning electron microscopy

**FIGURE 3:**
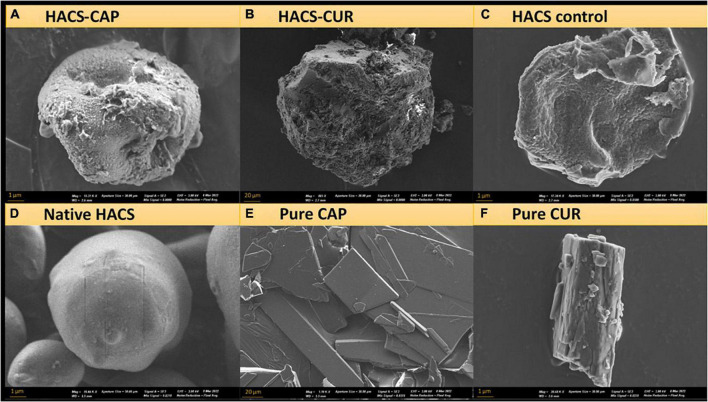
SEM images of **(A)** HACS-CAP, **(B)** HACS-CUR, **(C)** HACS control, **(D)** native HACS, **(E)** pure CAP, and **(F)** pure CUR.

Since inclusion appreciably alters the architecture of starch, surface morphology of HACS-CUR, HACS-CAP complexes (pure CUR, CAP, and HACS as controls) were investigated by scanning electron microscope (SEM) and selected micrographs are shown in Figure 3. These findings show that processing HACS into processed HACS, HACS-CUR or HACS-CAP led to the formation of less regular spheroids compared to the native HACS (Figure 3D). Moreover, these structures were distinctly different from the pure CAP or CUR samples (Figures 3E,F) that yielded typical crystallites. Comparison of the various processed HACS spheroids (Figures 3A–C) reveals the presence of CUR or CAP led to the formation of better-defined particulates, which concurs with the higher crystallinity (Table 1) measured for such samples. This overall findings indicate formation of particulate architectures with rougher surfaces and depressions, coinciding with the literature on V-type complexes formed via alkaline and acid treatments (47, 53). Such fine differences in the surface of particulates may alter their susceptibility to digestive elements, such as enzymes, as shown to be the case for protein-polysaccharide nano-particles (54).

#### Particles size distribution and volume mean diameter (d_4,3_)

**FIGURE 4:**
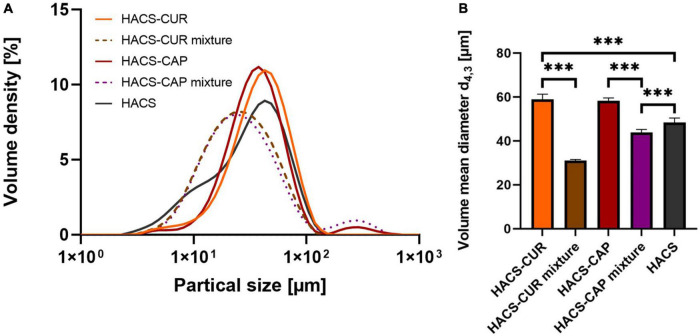
**(A)** Particle size distribution and **(B)** volume mean diameter of HACS-CUR and HACS-CAP, HACS control (empty) and physical mixtures. ***p < 0.001.

In addition to particle morphology and internal organization, colloid size distribution is of importance to the system’s colloidal stability as well as to digestion. Thus, HACS-phenol complexes were characterized by static light scattering (SLS) and the calculated particle size distribution (PSD) curves are shown in Figure 4. Overall, it was found that complexation led to an appreciable increase in particle sizes (Figure 4A) with significant (*p* < 0.05) increase in mean particle sizes (Figure 4B) of HACS-CUR and HACS-CAP compared to their counterpart physical mixtures or empty HACS. Despite slight deviations of the particle distributions of HACS-CUR and HACS-CAP complexes, no significant difference was observed in their mean volume diameter. In addition, HACS-CAP complexes registered a bi-modal size-distribution with a small sub-population of sizes exceeding 200 μm that may stem from unbound CAP assemblies or non-uniform interactions between CAP and starch components (amylopectin or amylose) yielding different architectures (mixtures of linear or helical), as also noted by others (24, 47).

### Ligand content in the complexes

**FIGURE 5:**
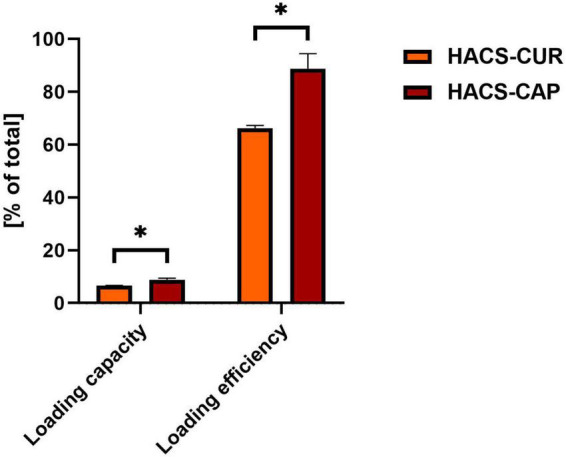
HACS complexes loading capacity and loading efficiency with CUR or CAP. *p < 0.05.

One key attribute of delivery vehicles is their ability to take on the cargo bioactive. To this end, loading capacity and loading efficiency were monitored for HACS-CAP and HACS-CUR complexes. Results obtained for these determinations are given in Figure 5. It can be seen that a high loading efficiency was obtained for HACS-CAP complex (88.77 ± 5.7%). Contrary, CUR entrapment was found to be significantly (*p* < 0.05) lower in both loading capacity and loading efficiency, yet values for CUR entrapment were still viable and found to be 6.63 ± 0.09% and 66.3 ± 0.1%, respectively. Interestingly, this overall trend concurs with the DSC findings (Figure 2) that demonstrate CAP is entrapped at a higher ratio than CUR. One possible explanation for such differences can be the varying physicochemical properties of CAP and CUR (e.g., logP and pH sensitivity) which mandated tweaking the production protocol and the use of milder pH conditions for entrapment of CUR. As detailed in the methods section, complex formation was done under milder conditions for CUR which under these conditions it is believed that amylose coils are more closed rendering them less accessible for entrapment.

### Evaluating the digestive performance of high amylose corn starch-phenol complexes

The functionality of HACS-phenol complexes is acted out during digestion, therefore, all complexes were studied for their potential digestive fate using a semi-dynamic GI model. This included monitoring the starch degradation as well as the release of the payload.

#### Starch degradation evaluation by dinitrosalicylic (DNS) acid assay

**FIGURE 6:**
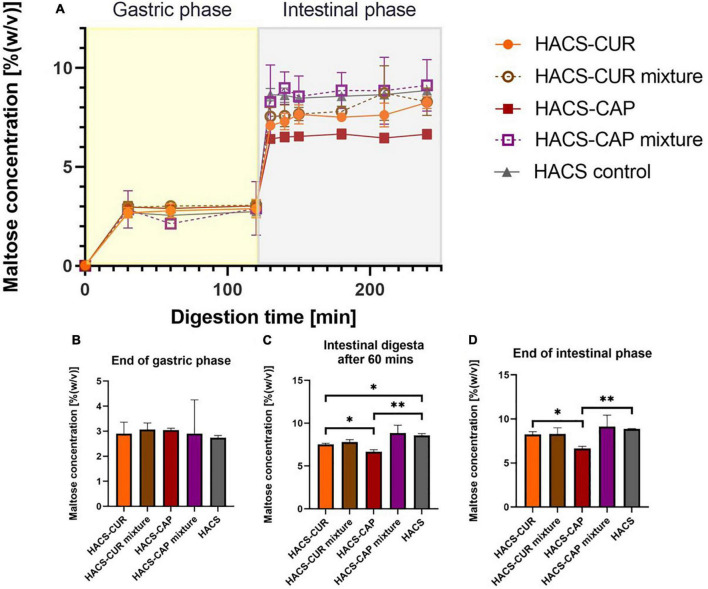
**(A)** Starch hydrolysis as maltose concentration in an adult in vitro digestion model. Bar charts can be seen at specific time points: **(B)** at the end of gastric phase, **(C)** after 60 min in the intestinal phase, and **(D)** at the end of intestinal phase. *p < 0.05, **p < 0.01.

Monitoring of maltose formation during the digestion of HACS-phenol complexes was performed as an indicator for starch breakdown and relevant results are shown in Figure 6. As can be seen in Figure 6A deviations in starch degradation between samples were markedly observed during the intestinal phase. Throughout gastric digestion no significant differences were observed (Figure 6B), however, during 60 min of intestinal digestion the presence of complexed CAP or CUR significantly (*p* < 0.05) attenuated HACS degradation compared to the empty HACS or the corresponding physical mixtures (Figure 6C). Yet, at the end of the intestinal phase (Figure 6D) only the degradation of HACS-CAP remained significantly (*p* < 0.05) lower. In the case of HACS-CUR, no significant difference was observed between HACS-CUR and HACS. Again, one can hypothesize that the lower entrapment of CUR hinders its impact on the digestion of HACS and contrary to CAP which is entrapped at a higher ratio. Overall, interactions of HACS with the ligands (CAP or CUR) seems to induce formation of structures with higher crystallinity (Table 1) which in turn may increase resistance to enzymatic degradation and explain the findings herein. Interestingly, the observed toning down of starch breakdown can offer another possible benefit in reducing bioaccessibility of glucose perhaps reducing glycemic responses, however, this notion must be ascertained experimentally.

#### Controlled release of payload under digestive conditions

**FIGURE 7:**
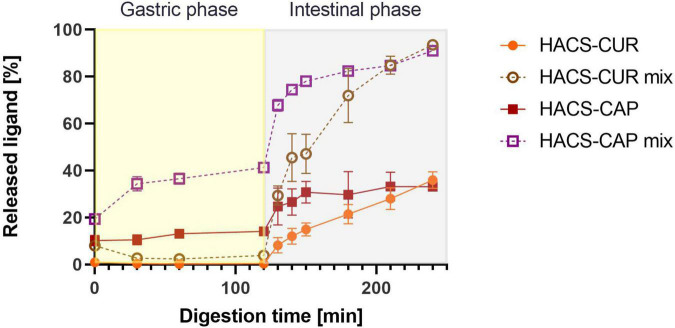
Release profiles of CUR and CAP in an adult *in vitro* digestion model.

Besides the degradation of the starch carrier, the release of the payload under the conditions of the gut is an important determinant of the system’s performance. Thus, the release of CAP or CUR under simulated GI condition was monitored and the results are shown in Figure 7. First, complexation of CAP or CUR with HACS was found to retain both ligands throughout the gastro-intestine better than their physical mixtures. In the case of HACS-CAP, delayed release was observed with only 35.98 ± 3.49% released after 2 h of intestinal digestion. Contrary, CUR exhibited good retention under gastric conditions possibly due to its poor solubility under gastric conditions. However, this was followed by sustained CUR release from HACS-CUR under the duodenal conditions up to 33.14 ± 0.37% after 2 h unlike 91.04 ± 0.98% released from the corresponding physical mixture. From the mechanistic point of view, one can suggest that the observed payload release patterns can arise from HACS degradation but also from particulate swelling leading to payload leaching. Specifically, the findings herein suggest complexation with HACS can sustain CAP and CUR release in the intestine with possible further payload release downstream in the small intestine or even the colon.

**FIGURE 8:**
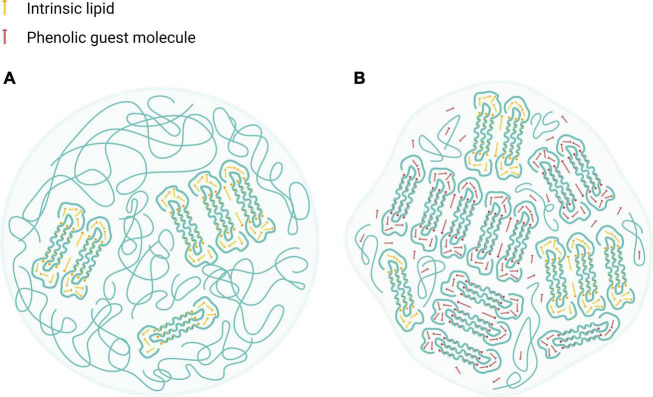
Schematic illustration of HACS particulate architectures in which crystalline V-amylose assemblies are interspaced by amorphous regions of amylose and amylopectin. **(A)** HACS colloid entrapping intrinsic moieties (marked as yellow rods). **(B)** HACS colloid entrapping intrinsic moieties (marked as yellow rods) and the encapsulated phenolic guest molecules (marked as red rods). Such colloids may further aggregates to form larger entities. Created with BioRender.com.

Altogether, HACS complexation with either CAP or CUR seems to enhance crystalline structures in the starch particulates, probably organized in lamellar assemblies, as suggested previously (12, 42, 47). Such lamellae are also found in the native HACS due the formation of amylose inclusion complexes that entrap intrinsic lipids that are naturally found inside the granules (∼1%) (55). Based on the findings herein, we suggest that native or processed HACS granules may contain low amounts of lamellar structures with crystalline regions while HACS complexes have higher levels of crystalline regions which also affect the overall particulate shape, as can be schematically seen in Figure 8. In fact, the alkaline and acid treatment of HACS in the presence of a ligand, induces V-amylose formation in starch spherulites where it is organized in nano and meso-scale lamellae that contribute to the crystalline structure of the formed spherulites, as also suggested by other studies (12, 47). Thus, HACS complexation with the guest phenolic compounds affects various dimensions of starch spherulites which in turn affect starch digestibility and the release of the guest molecules in the gut.

## Conclusion

Delivery of bioactive phenolic compounds is a scientific and technological challenge that can be tackled by rationally designed polysaccharide delivery vehicles, like V-amylose inclusion complexes. This study investigated the use of HACS to fabricate HACS-phenol inclusion complexes and evaluate their structure and possible digestive fate. XRD and DSC results indicate that the formation of inclusion complexes alters the supramolecular assembly of V-amylose as well as the properties of the formed colloids as shown by SLS and SEM. Interestingly, CAP was found to produce complexes with higher ligand content compared to CUR. In turn, these structural differences attenuated starch breakdown and sustained the intestinal release of the payloads. The observed differences between HACS-CAP and HACS-CUR complexes are believed to arise from differences in the fabrication process which leads to the formation of different starch architectures that in turn direct their digestive fate. Altogether, HACS-phenol inclusion complexes offer the possibility to effectively entrap phenolic ligands alongside attenuation of starch digestive breakdown. Moreover, both capsaicin and curcumin have distinct organoleptic properties that may be masked off by their inclusion in starch particulates, thus, offering an additional benefit of masking off adverse oral sensations. Thus, this work reinforces the possible applications for HACS as delivery vehicles for bioactive phenolics with sustained starch breakdown and perhaps a lowered glycemic index.

## Data availability statement

The raw data supporting the conclusions of this article will be made available by the authors, without undue reservation.

## Author contributions

HR: conceptualization, investigation, data curation, formal analysis, writing, and visualization. CS: methodology, investigation, writing – review and editing, and project administration. UL: supervision, project administration, conceptualization, resources, writing – review and editing, and funding acquisition. All authors contributed to the article and approved the submitted version.
