# Supplementary material for: Inclusion of phenolic bioactives in high amylose corn starch for gastro-intestinal delivery
Source: Front Nutr. 2022 Aug 25;9:981408. doi: 10.3389/fnut.2022.981408 (PMC9452773; doi:10.3389/fnut.2022.981408)
Supplement: Supplementary file 1 [file Data_Sheet_1.pdf]

Supplementary Material

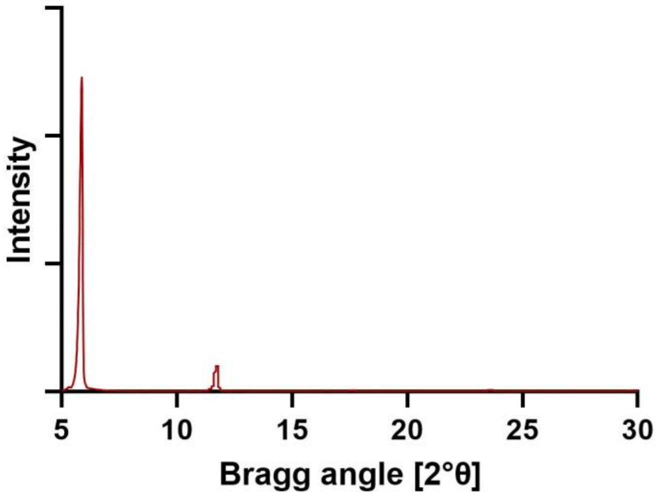

Supplementary Figure 1: X-ray diffraction patterns of pure capsaicin

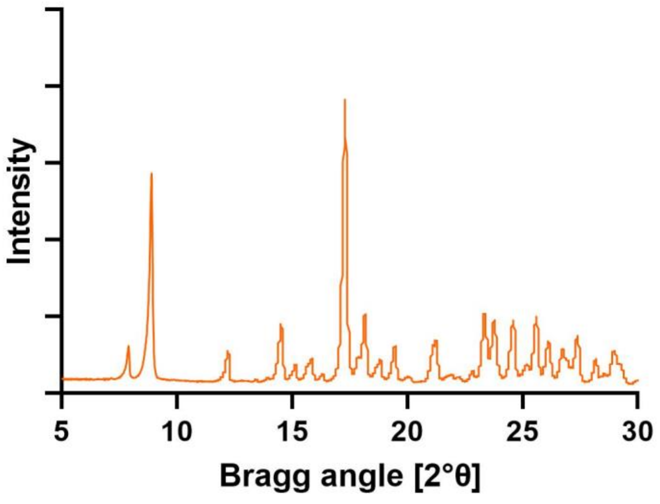

Supplementary Figure 2: X-ray diffraction patterns of pure curcumin
